# Mean radiant temperature from global-scale numerical weather prediction models

**DOI:** 10.1007/s00484-020-01900-5

**Published:** 2020-04-09

**Authors:** Claudia Di Napoli, Robin J. Hogan, Florian Pappenberger

**Affiliations:** 1 European Centre for Medium-Range Weather Forecasts, Reading, UK; 2 University of Reading, Reading, UK

**Keywords:** Mean radiant temperature, Radiation, Numerical weather prediction, Human comfort, Validation

## Abstract

In human biometeorology, the estimation of mean radiant temperature (MRT) is generally considered challenging. This work presents a general framework to compute the MRT at the global scale for a human subject placed in an outdoor environment and irradiated by solar and thermal radiation both directly and diffusely. The proposed framework requires as input radiation fluxes computed by numerical weather prediction (NWP) models and generates as output gridded globe-wide maps of MRT. It also considers changes in the Sun’s position affecting radiation components when these are stored by NWP models as an accumulated-over-time quantity. The applicability of the framework was demonstrated using NWP reanalysis radiation data from the European Centre for Medium-Range Weather Forecasts. Mapped distributions of MRT were correspondingly computed at the global scale. Comparison against measurements from radiation monitoring stations showed a good agreement with NWP-based MRT (coefficient of determination greater than 0.88; average bias equal to 0.42 °C) suggesting its potential as a proxy for observations in application studies.

## Introduction

The mean radiant temperature (MRT) is considered the most problematic variable to estimate in the assessment of human biometeorological comfort (Kántor and Unger 2011). Nevertheless, together with air temperature, humidity and wind speed, it is essential to describe the thermo-physiological effects of the outdoor environment on the human heat balance and comfort. The MRT is a measure of the total radiation from the atmosphere and the ground (*radiant environment*) incident on an object from all directions. Rather than expressing this measure as a flux density, i.e. the amount of radiation incident on a surface, it is converted into a temperature via the Stefan–Boltzmann equation. For a human located in a given environment with a given posture and clothing, the MRT is defined as “that uniform temperature of a fictive black-body radiation enclosure (emission coefficient ε = 1) which would result in the same net radiation energy exchange with the subject as the actual, more complex radiation environment” (Kántor and Unger 2011).

As a critical physical quantity representing how human beings experience radiation, the MRT has been the subject of studies in a variety of disciplines, from urban planning to public health and climate change (Lindberg and Grimmond 2011; Lindberg et al. 2014; Thorsson et al. 2014; Lau et al. 2015). In biometeorology, the MRT is used to calculate thermal stress indices, i.e. multi-variate parameters describing the overall heat load experienced by the human body when attempting to maintain a thermal equilibrium with the surroundings. Thermal stress indices that require MRT as input parameter are, for instance, the wet-bulb globe temperature (WBGT), the physiological equivalent temperature (PET) and the universal thermal climate index (UTCI) (Budd 2008; Höppe 1999; Błażejczyk et al. 2013).

Different methods exist to estimate the MRT in outdoor settings (Thorsson et al. 2007; Krüger et al. 2014). The experimental method consists in measuring the MRT on field using instruments, such as pyranometers and globe thermometers, that can sample different radiation components in a three-dimensional environment. In most cases, however, measurements are not available and/or not provided with continuity. For long-term studies without direct measurements of radiation fluxes, the MRT can be derived using a theoretical method, i.e. models (Matzarakis et al. 2010 and references herein). Models currently used are based on standard meteorological measurements, i.e. air temperature, humidity and solar radiation, and make assumptions about radiation-related parameters, such as surface emissivity, albedo and transmittance, which are set to default values and treated as constant (Kántor and Unger 2011). In reality, however, these parameters are site-specific and change in time. Numerical weather prediction (NWP) models are able to take this variability into account.

NWP models are a mathematical representation of the atmospheric system based on established laws of physics (e.g. conservation of mass, energy and momentum) as well as observations. They provide reanalyses (i.e. historical data; Dee et al. 2015) and forecasts of various weather parameters such as temperature, moisture, cloud cover and fluxes as output. NWP outputs can be employed in several applications and the computation of MRT is one of them. High-resolution radiation outputs from NWP models, for instance, have been used to predict MRT in an urban setting (Leroyer et al. 2018).

Calculating MRT from NWP outputs offers several advantages. First, NWP outputs are the result of a coherent theoretical framework where physical processes related to radiation and its interaction with land and atmosphere, i.e. albedo, atmospheric turbidity and Earth’s curvature, are modelled. In the NWP model from the European Centre for Medium-Range Weather Forecasts (ECMWF), for example, radiation is computed from temperature, humidity, cloud and monthly-mean climatologies of aerosols and trace gases (Hogan and Bozzo 2018). Second, NWP outputs are represented over a grid with each cell being the average value of a given parameter at the corresponding location. Differently from station data, they thus provide a spatially complete depiction of the atmospheric environment over different spatial domains, from the local to the global. Computing the MRT from NWP outputs generates therefore a gridded information map of radiation contribution to thermal comfort within a consistent and scientifically robust model.

Using NWP outputs also presents some challenges. One challenge is related to the accuracy of the NWP model. Model dynamics, physical parameterization, resolution, initial state and boundary conditions are sources of uncertainty in NWP outputs (Ehrendorfer 1997). Modelling cloud fraction and cloud types, for instance, is difficult and makes predicting radiation still a demanding task although significant improvements have been achieved (Hogan et al. 2009; Hogan and Bozzo 2018). Another challenge is represented by how outputs are stored in the NWP model. In the ECMWF NWP model, for example, radiation is stored at regular intervals (typically every 1 or 3 h) as an accumulated quantity since the start of the forecast. This offers an efficient way to compute the radiation transferred over a given time interval (e.g. a day) directly from archived quantities without having to store the outputs at every model step and average them. On the other side, however, it requires the movement of the Sun during the accumulation period to be accounted for (Hogan and Hirahara 2016). This is of particular importance because the Sun, as the primary source of energy for the atmospheric system, strongly influences the radiant environment with its position. In sunny conditions, for example, the radiant environment is dominated by direct solar radiation and this translates into a MRT up to 30 °C higher than air temperature (Jendritzky et al. 2000). These two aspects—radiation accumulated over a time period and the Sun’s position over the same accumulation period—have made problematic so far to compute MRT from NWP outputs stored as in the ECMWF NWP model.

The aim of the present study is threefold. First, it proposes a general framework to compute MRT from NWP radiation outputs by accounting for changes in the Sun’s position during the model accumulation time. Second, using outputs from the ECMWF NWP model, it provides the first mapped distribution of the MRT at the global scale by applying the proposed framework. Third, it validates the potential of NWP-based MRT as a proxy for station-based MRT via a statistical assessment against measurements from radiation monitoring stations.

## Data and methods

### Mean radiant temperature—the general equation

MRT is calculated by dividing the entire surroundings of the human body into *n* isothermal surfaces that have surface temperatures *T*_*i*_ and emission coefficients *ε*_*i*_ (*i* = 1 to *n*). Each of the *n* surfaces emits thermal radiation according to the Stefan–Boltzmann’s law (${E}_i={\varepsilon}_i\ \sigma\ {T}_i^4$) and reflects diffusely solar radiation *D*_*i*_ (Fanger 1972; Jendritzky et al. 1990). Both *E*_*i*_ and *D*_*i*_ are weighted by “angle factors” *F*_*i*_ describing the solid angle proportion of each emitting/reflecting surface. MRT can be therefore expressed as (Fanger 1972; Jendritzky and Nübler 1981):

1 $$ \mathrm{MRT}={\left[\frac{1}{\sigma }{\sum}_{i=1}^n\left({E}_i+{\alpha}_{ir}\frac{D_i}{\varepsilon_p}\right){F}_i\right]}^{0.25} $$

where *σ* is the Stefan–Boltzmann constant (5.67 × 10^−8^ W/m^2^K^4^) and *ε*_*p*_ is the emissivity of the clothed human body (standard value 0.97). *D*_*i*_ comprises the diffuse solar radiation (from the sky) and the diffusely reflected global radiation, whereas *α*_*ir*_ is the absorption coefficient of the body surface area irradiated by solar radiation (standard value 0.7). MRT is incremented to MRT* if direct solar radiation is also present (Jendritzky et al. 1990):

2 $$ {\mathrm{MRT}}^{\ast }={\left[{\mathrm{MRT}}^4+\frac{f_p{a}_{ir}{I}^{\ast }}{\left({\varepsilon}_p\sigma \right)}\right]}^{0.25} $$

where *I*^∗^ is the radiation intensity of the Sun on a surface perpendicular to the incident radiation direction. The surface projection factor *f*_*p*_ represents the portion of body surface exposed to direct solar radiation. It is a function of the incident radiation direction and the body posture (Jendritzky et al. 1990). In the next sections, each of the radiation parameters *E*_*i*_, *D*_*i*_ and *I*^∗^will be defined in terms of the radiation components computed by the ECMWF NWP model.

### Radiation in a NWP model

The ECMWF NWP model provides both solar and thermal radiation quantities (Hogan and Bozzo 2018). *Solar* (or *short-wave*) refers to radiation emitted by the Sun, then scattered, absorbed or transmitted by the atmosphere and reflected or absorbed by the surface. *Thermal* (or *long-wave*) refers to radiation emitted and absorbed by the surface or by gases, clouds and particles within the atmosphere.

As described in the “Introduction”, radiation quantities are stored as *accumulated* fluxes, i.e. as the energy that has passed through a square metre of a flat horizontal plane (units of J m^−2^ or W m^−2^ s) since the start of the relevant forecast. Global NWP models are generally configured with variables output at distinct time steps, e.g. every Δ*t* = 1 or 3 h. This is much longer than the internal model time step, which is currently 7.5 min in the highest-resolution forecasts run at ECMWF. A mean radiation flux (*accumulated mean*) is obtained by retrieving two accumulated fluxes at *t*_1_=*t* ~and *t*_2_ = *t* ~  + *∆t* (where *t̃* is the time of the start of the average), taking the difference and dividing by the number of seconds in Δ*t*.

In the determination of MRT, the focus is on the radiative heat exchange between the human body and its environment. Therefore, *surface* fluxes are considered, i.e. fluxes that are reported to the ground level according to the model’s representation of orography.

**Fig. 1:**
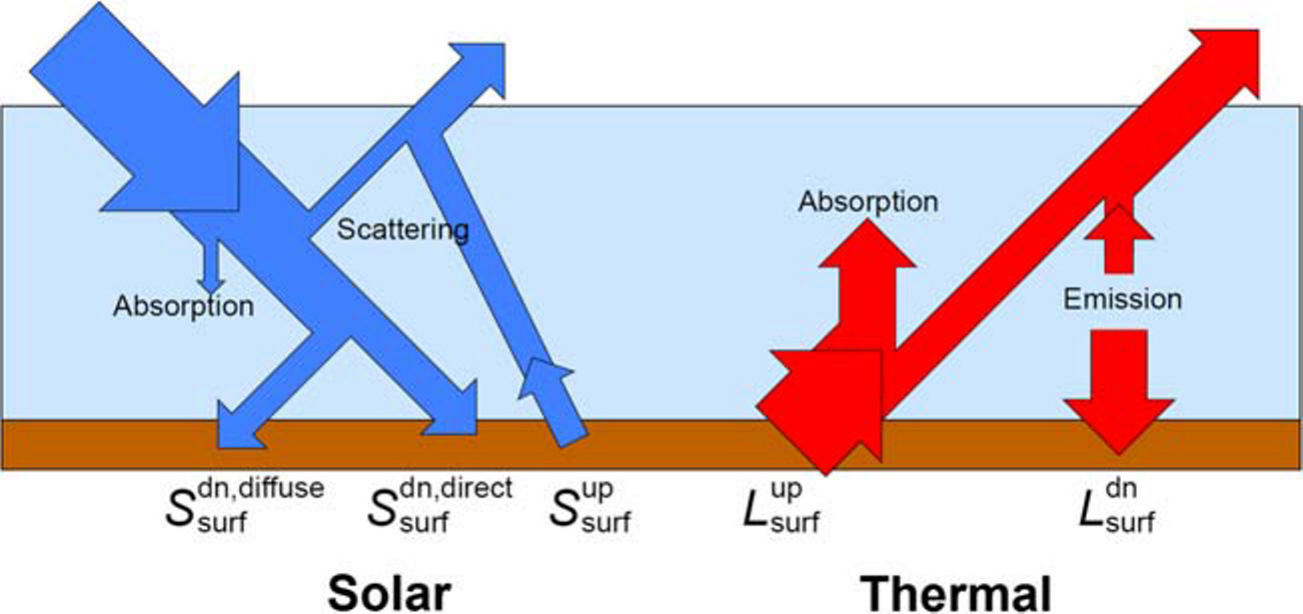
Schematic of the solar and thermal radiative energy flows in the atmosphere. The symbols for upwelling and downwelling fluxes at the surface are explained in Table 1. Adapted from Hogan (2015)

The MRT can be estimated from the ECMWF NWP model by using the accumulated means of the radiation components represented in Fig. 1. These are the surface solar radiation downwards ${S}_{\mathrm{surf}}^{\mathrm{dn}}$, the surface net (down minus up) solar radiation ${S}_{\mathrm{surf}}^{\mathrm{net}}$, the direct solar radiation at the surface ${S}_{\mathrm{surf}}^{\mathrm{dn},\mathrm{direct}}$, the surface thermal radiation downwards ${L}_{\mathrm{surf}}^{\mathrm{dn}}$ and the surface net thermal radiation ${L}_{\mathrm{surf}}^{\mathrm{net}}$. These are provided by ECMWF as both forecast outputs and atmospheric reanalysis products. In an atmospheric reanalysis, surface and atmospheric parameters are computed by combining the NWP model and quality-controlled observations in a dynamically consistent estimate of the past state of the atmosphere via an assimilation scheme (Poli 2012). ECMWF reanalysis radiation products are freely available at the Copernicus Climate Data Store (CDS, https://cds.climate.copernicus.eu/). For consistency, the nomenclature adopted in this study for the radiation components is the same as the ECMWF NWP model (Table 1).

**Table 1:** Surface fluxes used to compute the MRT from ECMWF NWP model outputs. The unit of measurement for corresponding accumulated means is watts per square metre. Symbols are as shown in Fig. 1. Note that downward and net fluxes are the only types of flux outputs from the ECMWF NWP model. As the model regards the energy entering the Earth’s atmosphere–surface system as positive, downward fluxes are considered positive and net fluxes refer to the difference between downward fluxes and upward fluxes (Hogan 2015). The upward flux may be trivially computed as the downward flux minus the net

Name	Symbol/equation
Surface solar radiation downwards	${S}_{\mathrm{surf}}^{\mathrm{dn}}={S}_{\mathrm{surf}}^{\mathrm{dn},\mathrm{direct}}+{S}_{\mathrm{surf}}^{\mathrm{dn},\mathrm{diffuse}}$
Surface net solar radiation	${S}_{\mathrm{surf}}^{\mathrm{net}}={S}_{\mathrm{surf}}^{\mathrm{dn}}-{S}_{\mathrm{surf}}^{\mathrm{up}}$
Direct solar radiation at the surface	${S}_{\mathrm{surf}}^{\mathrm{dn},\mathrm{direct}}$
Surface thermal radiation downwards	${L}_{\mathrm{surf}}^{\mathrm{dn}}$
Surface net thermal radiation	${L}_{\mathrm{surf}}^{\mathrm{net}}={L}_{\mathrm{surf}}^{\mathrm{dn}}-{L}_{\mathrm{surf}}^{\mathrm{up}}$

### Estimation of thermal radiation

The thermal radiation parameter *E*_*i*_ consists of two components: an atmospheric downwelling component and an upwelling component from the ground. In Fig. 1, they correspond to ${L}_{\mathrm{surf}}^{\mathrm{dn}}$ and ${L}_{\mathrm{surf}}^{\mathrm{up}}$, respectively. In the ECMWF NWP model, the upwelling component ${L}_{\mathrm{surf}}^{\mathrm{up}}$ can be retrieved from the surface thermal radiation downwards and the surface net thermal radiation as (Table 1)

3 $$ {L}_{\mathrm{surf}}^{\mathrm{up}}={L}_{\mathrm{surf}}^{\mathrm{dn}}-{L}_{\mathrm{surf}}^{\mathrm{net}} $$

### Estimation of solar radiation

The solar radiation flux consists of two components: a diffuse component and a direct component. The diffuse component, previously indicated as *D*_*i*_, is the sum of the isotropic diffuse solar radiation flux and the surface-reflected solar radiation flux. In Fig. 1, they correspond to ${S}_{\mathrm{surf}}^{\mathrm{dn},\mathrm{diffuse}}$ and ${S}_{\mathrm{surf}}^{\mathrm{up}}$, respectively. They can be computed from the surface solar radiation downwards, the surface net solar radiation and the direct solar radiation at the surface components as follows (Table 1):

4 $$ {S}_{\mathrm{surf}}^{\mathrm{dn},\mathrm{diffuse}}={S}_{\mathrm{surf}}^{\mathrm{dn}}-{S}_{\mathrm{surf}}^{\mathrm{dn},\mathrm{diffuse}} $$

5 $$ {S}_{\mathrm{surf}}^{\mathrm{up}}={S}_{\mathrm{surf}}^{\mathrm{dn}}-{S}_{\mathrm{surf}}^{\mathrm{net}} $$

The direct component *I*^∗^ can be obtained via the direct solar radiation at the surface ${S}_{\mathrm{surf}}^{\mathrm{dn},\mathrm{direct}}$ by converting the latter from a flux through a flat horizontal plane (as it is provided by the ECMWF NWP model) to a flux through a plane oriented perpendicular to the incoming solar radiation (as it is requested in the MRT equation). This can be achieved by dividing ${S}_{\mathrm{surf}}^{\mathrm{dn},\mathrm{direct}}$ by the cosine of the solar zenith angle, cos*θ*_0_. Such a conversion is, however, not trivial. As previously explained, fluxes are stored as accumulated quantities over a period of time Δ*t*. During this period, the Sun’s position and thus the solar zenith angle changes. How ${S}_{\mathrm{surf}}^{\mathrm{dn},\mathrm{direct}}$ can be converted to a flux through a perpendicular plane by keeping into account both the radiation accumulation and the solar zenith angle evolution over time is explained in the next sections.

#### Solar coordinates

The cosine of the solar zenith angle can be computed as (Woan 2000)

6 $$ \cos {\theta}_0=s\mathrm{in}\delta \sin \phi +\cos \delta\ \cos \phi\ \cos h $$

where *δ* is the solar declination angle, *ϕ* is the geographical latitude and *h* is the hour angle in the local solar time. In an equatorial coordinate system, *δ* and *h* specify the position of the Sun at a given date *JD* (Julian day number) and a given hour *hr*. If *g* is the corresponding angular fraction of the year in degrees

7 $$ g=\frac{360}{365.25}\left( JD+\frac{hr}{24}\right) $$

the solar declination angle may be computed as (Spencer 1971)

8 $$ \delta =\left(180/\pi \right)\times \left(0.006918-0.399912\ \cos (g)+0.070257\ \sin (g)-0.006758\ \cos (2g)+0.000907\ \sin (2g)-0.002697\ \cos (3g)+0.001480\ \sin (3g)\right) $$

The solar hour angle is given by (NOAA 1997)

9 $$ h=\left( hr-12\right)\cdot 15+\lambda + TC $$

where *λ* is the geographical longitude and *TC* is the time correction

10 $$ TC=0.004297+0.107029\ \cos (g)-1.837877\ \sin (g)-0.837378\ \cos (2g)-2.340475\ \sin (2g) $$

When the Sun is below the horizon, cos*θ*_0_ is equal to zero.

#### Average daytime cosine of the solar zenith angle

The direct component *I*^∗^ could be calculated by dividing the time average of ${S}_{\mathrm{surf}}^{\mathrm{dn},\mathrm{direct}}$ by the time average of cos*θ*_0_, but when the accumulation time encompasses sunrise or sunset, this leads to I^*^ being overestimated. Hogan and Hirahara (2016) demonstrated in a similar context that it is more appropriate to use the average *daytime* cosine of the solar zenith angle, $\overline{\cos {\theta}_0}$, i.e. the average cosine of the solar zenith angle for the sunlit part of the interval Δ*t* between the two archiving times *t*_1_ and *t*_2_. They found that when $\overline{\cos {\theta}_0}$ is used, biases due to infrequent radiation calculations in some weather and climate models (such as a temperature overestimate in the stratosphere) are mitigated. In their work, the authors also provide the mathematical procedure for a correct computation of $\overline{\cos {\theta}_0}$ and it is here reported. First, the hour angle at either sunrise (when the negative value is taken) or sunset (when the positive value is taken) is computed via the Sunrise Equation from solar declination and geographical latitude

11 $$ \cos {h}_0=-\tan \delta \tan \phi $$

Second, the model time steps *t*_1_ and *t*_2_ are converted to hour angles *h*_1_ and *h*_2_, and compared to the values at sunrise and sunset to obtain the time interval *h*_min_ to *h*_max_ when the Sun is above the horizon. The average daytime cosine of the solar zenith angle is found by integrating (6) with respect to *h* in this time interval, which yields

12 $$ \overline{\cos {\theta}_0}=\sin \delta \sin \phi +\frac{1}{h_{\mathrm{max}}-{h}_{\mathrm{min}}}\cos \delta \cos \phi \left(\sin {h}_{\mathrm{max}}-\sin {h}_{\mathrm{min}}\right) $$

The output is a global map at the selected model time step showing the distribution of $\overline{\cos {\theta}_0}$ across the whole latitude–longitude range.

#### Projecting the direct component

The direct component *I*^∗^ is computed by dividing the direct solar radiation at the surface component by $\overline{\cos {\theta}_0}$:

13 $$ {I}^{\ast }=\frac{S_{\mathrm{surf}}^{\mathrm{dn},\mathrm{direct}}}{\overline{\cos {\theta}_0}\ } $$

In order to avoid division by zero, ${S}_{\mathrm{surf}}^{\mathrm{dn},\mathrm{direct}}$ is projected into a perpendicular plane at $\overline{\cos {\theta}_0}$ strictly greater than zero.

### Computing NWP-based mean radiant temperature

Using the nomenclature introduced in this section, Eq. (2) for the computation of MRT from NWP radiation outputs can be rewritten as (Staiger and Matzarakis 2010):

14 $$ {\mathrm{MRT}}^{\ast }={\left\{\frac{1}{\sigma}\left[{f}_a\ {L}_{\mathrm{surf}}^{\mathrm{dn}}+{f}_a\ {L}_{\mathrm{surf}}^{\mathrm{up}}+\frac{a_{ir}}{\varepsilon_p}\left({f}_a\ {S}_{\mathrm{surf}}^{\mathrm{dn},\mathrm{diffuse}}+{f}_a\ {S}_{\mathrm{surf}}^{\mathrm{up}}+{f}_p\ {I}^{\ast}\right)\right]\right\}}^{0.25} $$

with the angle factors *F*_*i*_ set equal to *f*_*a*_ = 0.5. For most applications at the macro-scale (i.e. beyond urban level), a limitation to two angle factors, each of value 0.5, can be sufficient. This corresponds to considering the surroundings of a human body as an unobstructed, flat site made of a lower hemisphere (ground) and an upper hemisphere (sky) only. The surface projection factor *f*_*p*_ can be determined from the solar elevation angle *γ* via different equations (Holmer et al. 2018). We choose to express *f*_*p*_ for a rotationally symmetric standing or walking person via a regression equation that had been based on photographs of a real person (*γ* in degrees; Jendritzky et al. 1990; VDI 1998)

15 $$ {f}_p=0.308\ \cos \left(\gamma \left(0.998-{\gamma}^2/50000\right)\right) $$

and used for human biometeorological comfort applications (Błażejczyk et al. 1993).

The solar elevation angle is complementary to the solar zenith angle and can therefore be expressed as

16 $$ \gamma =90{}^{\circ}-{\theta}_0 $$

To demonstrate the applicability of the framework presented in this study, MRT was computed from the model outputs of ERA5, the latest ECMWF global atmospheric reanalysis (Hersbach et al. 2019). ERA5 data are provided at the global scale on a regular grid at 0.25° × 0.25° resolution (~ 31 km) and currently span from 1 January 1979 to the present date. Radiation fields listed in Table 1 were retrieved from the ERA5 database at different model time steps and transformed into accumulated means. Equations (3), (4) and (5) were used to determine ${L}_{\mathrm{surf}}^{\mathrm{up}}$, ${S}_{\mathrm{surf}}^{\mathrm{dn},\mathrm{diffuse}}$ and ${S}_{\mathrm{surf}}^{\mathrm{up}}$. As far as the direct solar component is concerned, global maps of the average daytime cosine of the solar zenith angle were first computed across the whole latitude–longitude range and at the same time steps at which NWP radiation outputs have been retrieved. From these maps, ${S}_{\mathrm{surf}}^{\mathrm{dn},\mathrm{direct}}$ was then projected into *I*^∗^ (Eq. 13) and the surface projection factor determined via the solar elevation angle (Eq. 16). Once all the parameters needed in Eq. (14) were determined, global maps of NWP-based MRT were calculated.

### Validation against station data

The quality of NWP-based MRT was statistically assessed against the MRT calculated using local radiation data from eleven monitoring stations of the World Radiation Monitoring Center–Baseline Surface Radiation Network (WRMC–BSRN, Driemel et al. 2018). Stations were selected based on their ability to measure the observed counterparts of the NWP radiation fields used to compute MRT, namely direct normal radiation, diffuse radiation, upwelling/downwelling thermal radiation and upwelling solar radiation. Stations are located across the globe in sites characterized by different surface types and topography. These are listed in Table 2 together with the data periods available from the WRMC–BSRN database and used in this study.

**Table 2:** List of monitoring stations from the World Radiation Monitoring Center—Baseline Surface Radiation Network used to validate NWP-based radiation and MRT outputs. Stations have been selected based on their ability to measure and therefore provide all the observed counterparts of the NWP radiation fields used to compute MRT. The number of months for which station data are available is reported in brackets in the corresponding column. Adapted from https://www.pangaea.de/ddi?request=bsrn/BSRNEvent&format=html&title=BSRN+Stations and https://dataportals.pangaea.de/bsrn/

Station	Short name	Location	Latitude [°]	Longitude [°]	Elevation [m]	Surface	Topography	Available data
Alert	ALE	Lincoln Sea	82.49	− 62.42	127	Tundra	Hilly, rural	2004–2014 [119]
Barrow	BAR	Alaska, USA	71.323	− 156.607	8	Tundra	Flat, rural	1992–2017 [303]
Cabauw	CAB	The Netherlands	51.9711	4.9267	0	Grass	Flat, rural	2013–2018 [63]
Cape Baranova	CAP	Russia	79.27	101.75	–	–	–	2016 [12]
Gobabeb	GOB	Namib Desert, Namibia	− 23.5614	15.042	407	Desert	Flat, rural	2012–2018 [75]
Izaña	IZA	Tenerife, Spain	28.3093	− 16.4993	2372.9	Rock	Mountain top, rural	2016–2018 [25]
Ny-Ålesund	NYA	Ny-Ålesund, Spitsbergen	78.925	11.93	11	Tundra	Mountain valley, rural	1992–2018 [311]
Payerne	PAY	Switzerland	46.815	6.944	491	Cultivated	Hilly, rural	1992–2018 [315]
Tateno	TAT	Japan	36.0581	140.1258	25	Grass	Flat, urban	1996–2018 [274]
Tiksi	TIK	Siberia, Russia	71.5862	128.9188	48	Tundra	Flat, rural	2011–2018 [84]
Toravere	TOR	Estonia	58.254	26.462	70	Grass	Flat, rural	1999–2018 [240]

Both NWP-based and observed radiation fields, corresponding MRTs and mean radiant fluxes (MRF = *ε*_*p*_ *σ* MRT^4^) were first averaged in 3-h periods as a better agreement between models and observations had been demonstrated when data are averaged in time (Hogan et al. 2009). NWP-based quantities (*m*_*i*_) were then compared to observations (*o*_*i*_) using three metrics: coefficient of determination [*R*^2^=cov(*m*_*i*_,*o*_*i*_)^2^/var(*m*_*i*_)/var(*o*_*i*_)], bias [*Σ*(*m*_*i*_ − *o*_*i*_)/*n*] and root mean square error [RMSE = sqrt(*Σ*(*m*_*i*_ − *o*_*i*_)^2^/*n*)]. The *R*^2^ measures how well NWP-based values are correlated with observed values. The bias measures systematic errors, i.e. how the average NWP-based value compares to the average observed value. The RMSE measures how much NWP-based values deviate, on average, from observed values.

## Results

**Fig. 2:**
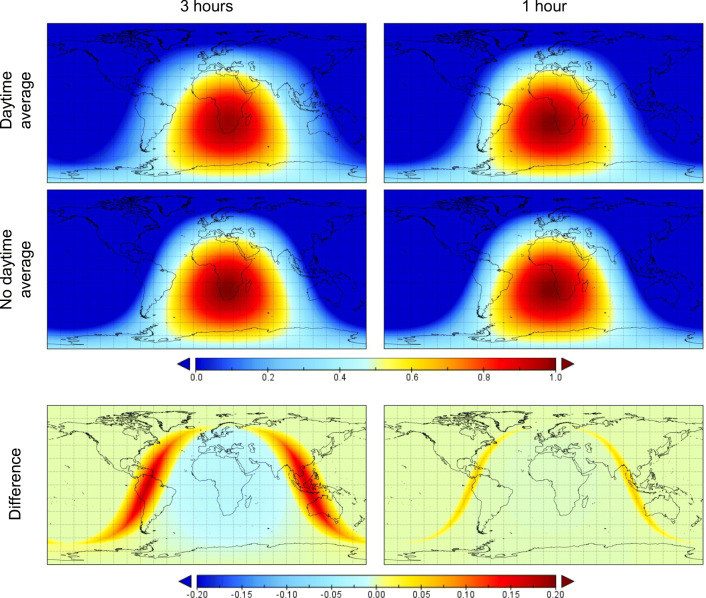
Global maps of the cosine of the solar zenith angle calculated with and without daytime averaging (first and second row, respectively) and corresponding difference (third row) for 1st January 2018 at 12UTC. They apply to a NWP model where the radiation scheme is called every 3 h and 1 h (left and right, respectively)

A proper globe-wide computation of MRT from NWP outputs relies on the appropriate modelling of the Sun’s position across the model time periods over which the radiant components contributing to MRT are accumulated. Figure 2 shows the mapped distributions of the cosine of the solar zenith angle with and without averaging only over the sunlit part of the time interval (first and second row, respectively) at the global scale. Values were computed for a representative date and time—1st January 2018 at 12UTC (universal time coordinated)—and apply to the calculation of MRT from ECMWF NWP model outputs for accumulation time intervals Δ*t* equal to 3 h or 1 h (left and right, respectively). The main effect of averaging the cosine of the solar zenith angle over the sunlit part of the radiation time step is observed at the locations where the distributions equal zero, i.e. where the day starts and ends at the selected model time step. When daytime averaging is applied, the cosine of the solar zenith angle reaches zero over a wider latitude/longitude gradient than when it is not applied. This is because the averaging is performed over the entire time interval, even when the Sun is below the horizon. Specifically, it adds Δ*t*/2 hours to each end of the day, i.e. before the Sun rises and after the Sun sets. As a consequence, the gradient is larger at Δ*t* equal to 3 h than 1 h. Another effect of averaging is to lower the value of the cosine of the solar zenith angle in the locations in daylight. This is also more evident at Δ*t* equal to 3 h.

**Fig. 3:**
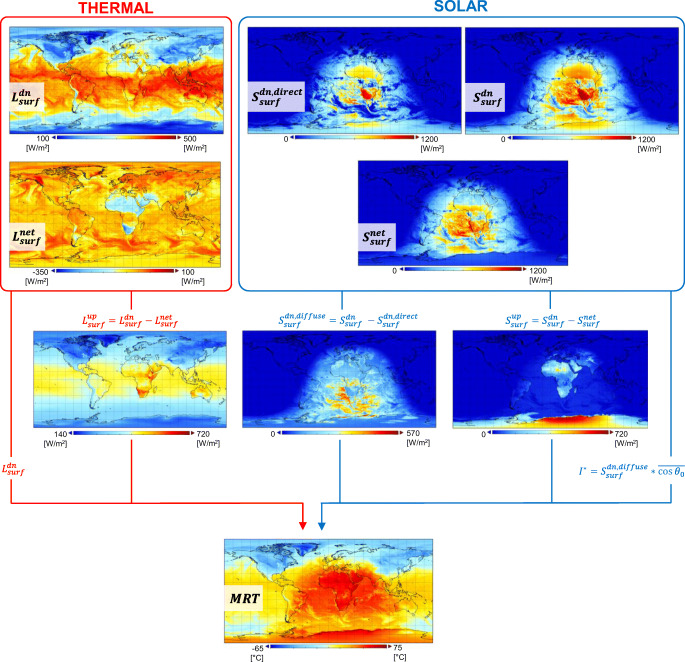
Application of radiation outputs from the ECMWF NWP model to the theoretical framework presented in the paper and the MRT field generated as output (12UTC)

Figure 3 illustrates an example of NWP-based MRT field as calculated by applying the theoretical framework herein presented to ECMWF ERA5 radiation outputs retrieved at 1-h steps and with the cosine of the solar zenith angle computed over Δ*t* = 1 h. It is worth noting the distribution of solar radiation which shows a strong dependence on the Sun’s position and the very high upwelling component from the surface ${S}_{\mathrm{surf}}^{\mathrm{up}}$ in Antarctica which is due to ice cover and associated high albedo. This influences how MRT is distributed over the globe, with higher values located in the sunlit part of the selected model time step.

**Fig. 4:**
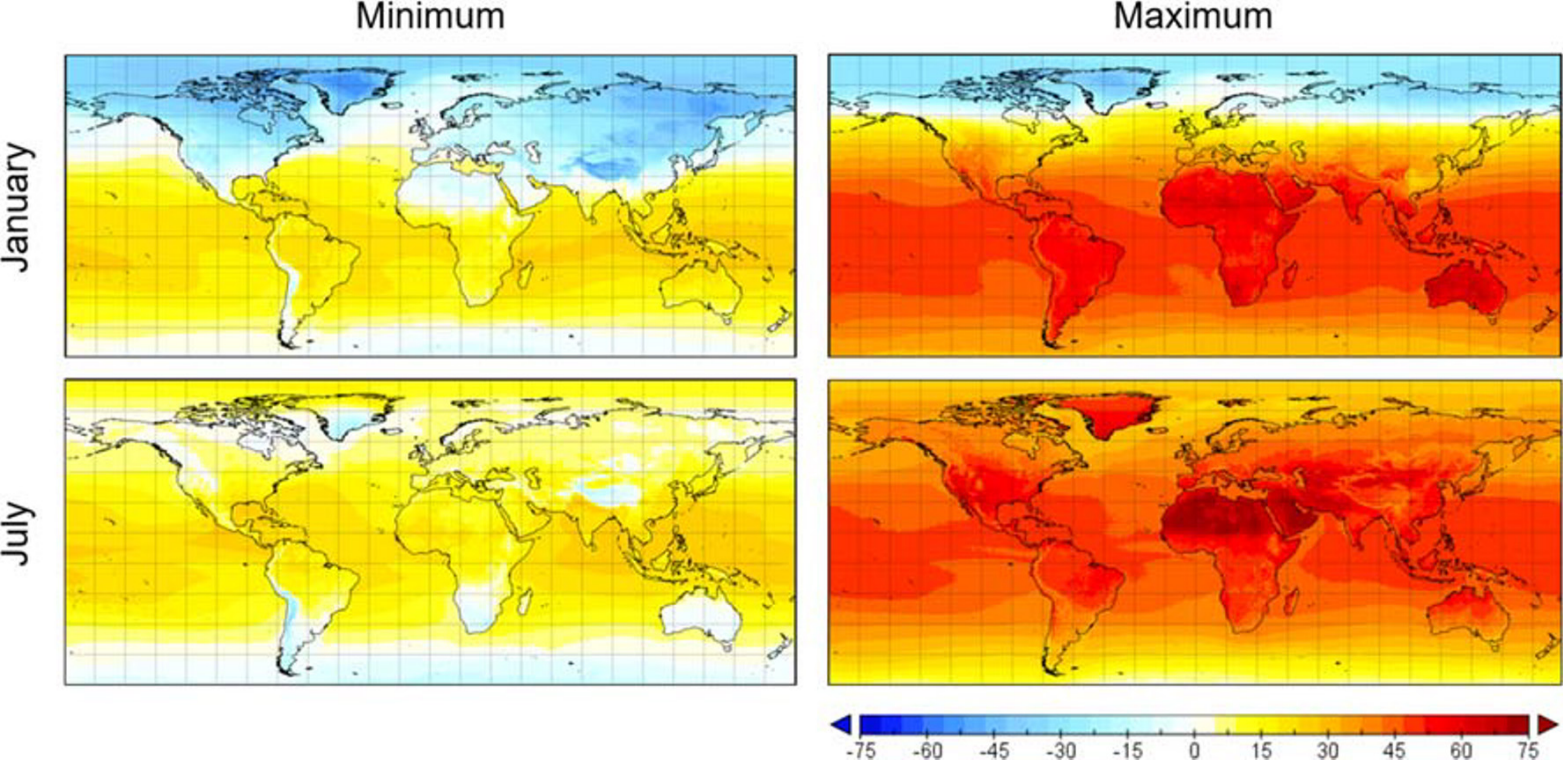
Climatology of average daily minimum and maximum MRT (in °C) for the months of January and July as calculated from ECMWF NWP reanalysis outputs, 1-h time step, period 1981–2010

A climatology of NWP-based MRT for the period 1981–2010 is shown in Fig. 4. Specifically, daily minimum and maximum MRTs computed from ECMWF ERA5 reanalysis (Δ*t* = 1 h) and averaged across the climatological period at two selected months, January and July, are represented. The MRT shows a clear seasonal pattern. The highest values characterize almost the whole of the southern hemisphere in January, the northern hemisphere in July and tropical regions in both months. In addition, there are significant regional variations, mainly related to surface type. For instance, maximum MRT gets close and above 70 °C in deserts. The low cloudiness and high albedo characterizing those areas increase the portion of the solar flux incident on the ground and reflected by it. With regard to minimum MRT, this is equal to or less than 28 °C, and it is due to the thermal component as the solar component is missing at nighttime.

**Fig. 5:**
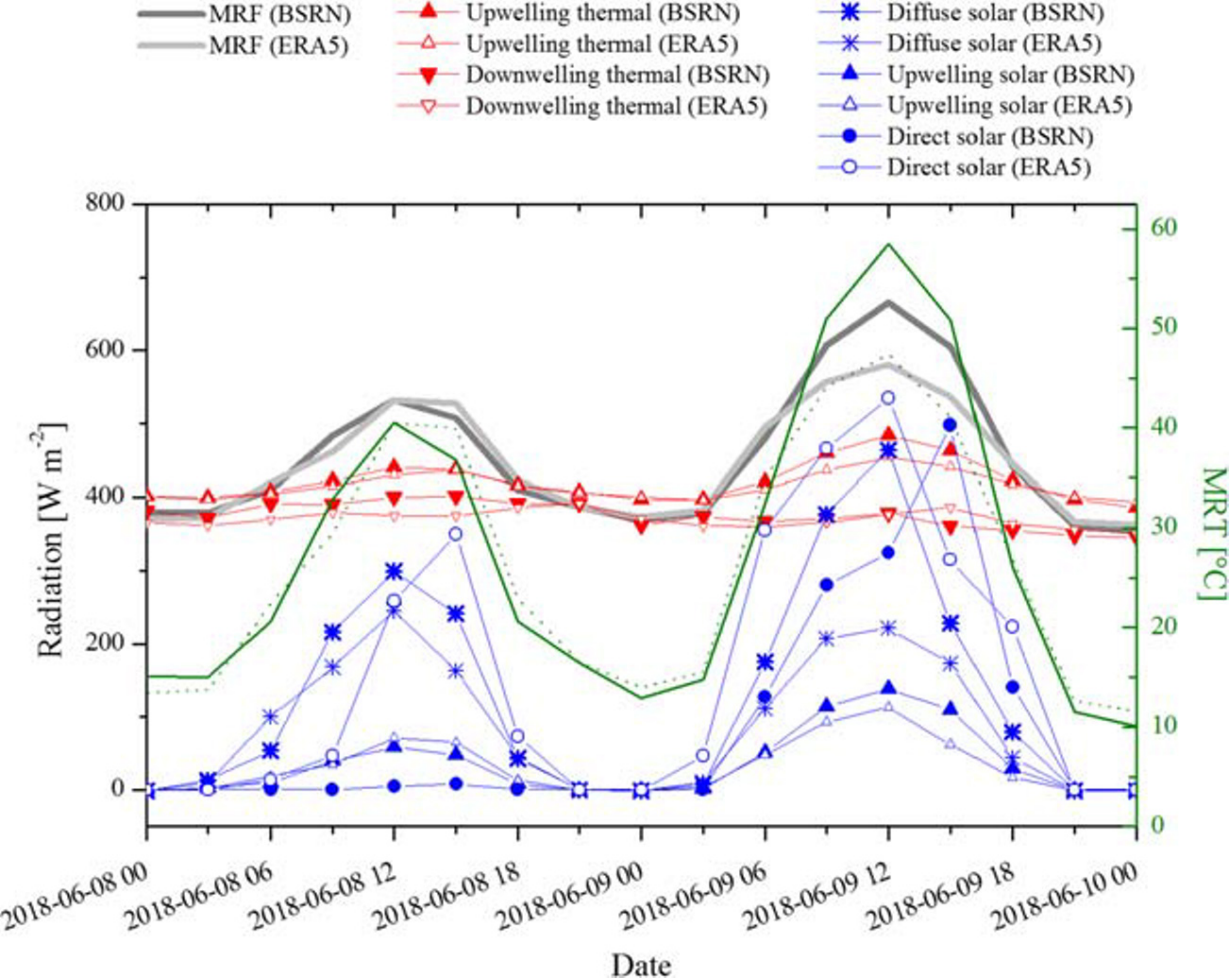
Temporal trends of mean radiant fluxes (MRF), thermal and solar radiation components as measured by the World Radiation Monitoring Center–Baseline Surface Radiation Network station in Cabauw on 8th and 9th June 2018. ERA5 values extracted from the corresponding grid cell for the same variables are also shown. MRT values calculated from observed and NWP-based data are shown as solid and dashed green lines, respectively

The temporal trend of both solar and thermal components, and their contribution to mean radiant flux and MRT is shown in Fig. 5. Values as measured by the WRMC–BSRN station in Cabauw are depicted during a 2-day sample period, 8th and 9th June 2018 together with ERA5-based values of the same variable extracted at the corresponding grid cell. All radiation components but the downwelling thermal component follow a distinctive diurnal cycle, with values higher in the daytime than in nighttime. Higher solar flux values are reached on 9th June when clear sky conditions were prevalent, compared to the previous day when overcast conditions dominated instead. This translates into a mean radiant flux and MRT that also shows a diurnal trend and have daytime values higher on 9th June than on 8th June. Figure 5 provides also a first insight on how NWP-based quantities compare to WRMC–BSRN observations. On 8th June, reanalysis data are in general similar to observed ones but the diffuse solar component (underestimated at 09, 12 and 15 UTC) and the direct solar component (overestimated at 09, 12, 15 and 18 UTC). The aggregative effect of all radiation components delivers MRT similar to observation-based MRT. On 9th June, the NWP-based diffuse solar radiation is still underestimated in the daytime. The observed direct solar radiation peaks at 15 UTC whereas the NWP-based counterpart shows consistently higher values across the first half of the day. The resulting NWP-based MRT underestimates the observed MRT at 9, 12 and 15 UTC.

**Fig. 6:**
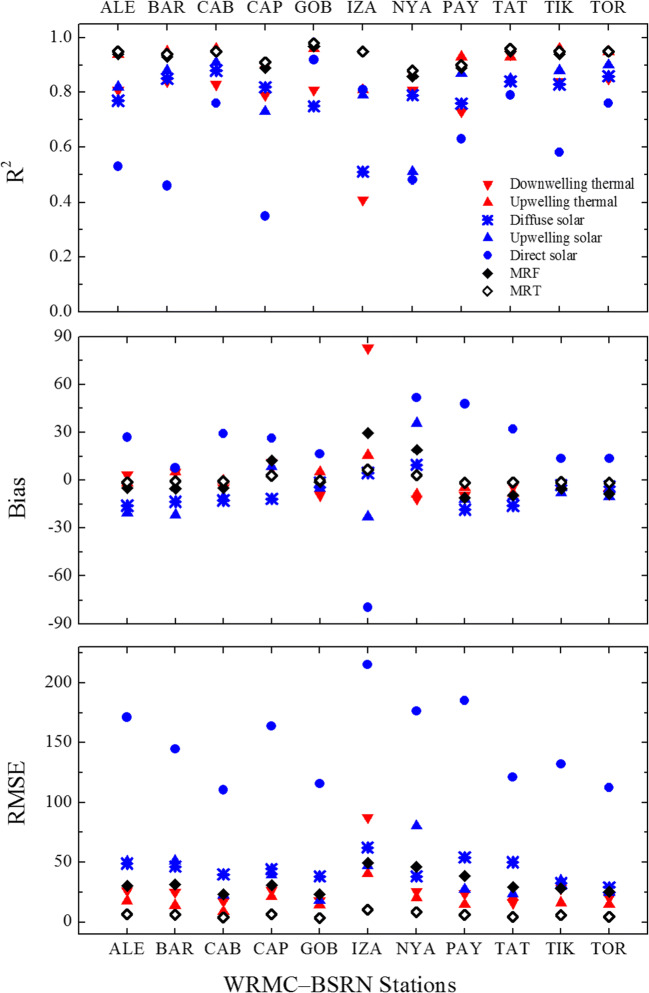
Validation of NWP-based radiation fields against corresponding measurements from eleven monitoring stations of the World Radiation Monitoring Center–Baseline Surface Radiation Network. Bias and RMSE are in degrees Celsius when referring to MRT and watts per square metre when referring to radiation fields and mean radiant fluxes (MRF)

The comprehensive validation of NWP-based quantities against corresponding WRMC–BSRN observations is summarized in Fig. 6. In general, a good agreement between NWP-based radiation fields and observed values can be found with *R*^2^ > 0.7, biases between −30 and 30 W/m^2^ and RMSE below 100 W/m^2^. NWP-based direct solar radiation is, however, characterized by a lower correspondence to observed direct solar radiation with 0.35 ≤ *R*^2^ ≤ 0.79 at most stations, biases up to 52 and −79 W/m^2^ and RMSE between 110 and 215 W/m^2^. In terms of stations, Izaña in Tenerife shows the highest associated uncertainties with the lowest *R*^2^ and the highest bias and RMSE. As far as mean radiant variables are concerned, a good overall agreement between NWP-based MRF and MRF calculated from WRMC–BSRN observations can be found with *R*^2^ ≥ 0.89, biases between −12 and 30 W/m^2^ and RMSE below 50 W/m^2^. This translates for MRT into good validation metrics, namely *R*^2^ ≥ 0.88, biases between −1.6 and 6.6 °C and RMSE less than 10 °C.

## Discussion

This study shows the result of computing globe-wide MRT from NWP outputs by taking into account the accumulated means of radiation input components and the change of the Sun’s position over the corresponding interval.

NWP-based radiation components from ECMWF ERA5 reanalysis dataset and the corresponding MRT were evaluated against the same quantities measured by a network of monitoring stations across the globe (WRMC–BSRN). Validation metrics show a good correspondence between modelled and observed quantities for upwelling and downwelling thermal radiation and for diffuse and upwelling solar radiation. Modelled direct solar radiation compared instead less well to its observed counterpart.

This is mainly related to the NWP skill of predicting cloudiness, specifically the timing, placement and type of clouds. The cloud cover and occurrence of a boundary layer and mid-level clouds, for instance, have been shown to be underestimated (Illingworth et al. 2007; Hogan et al. 2009). With more overcast conditions observed than modelled, systematic but partially compensating surface radiation errors exist, such as a positive bias in solar radiation (Ahlgrimm and Forbes 2014). This is in agreement with the results from the present study. To improve the accuracy of radiation calculations, the radiation schemes of almost all NWP climate models employ “delta-Eddington” scaling (Joseph et al. 1976), whereby some fraction of forward-scattered radiation is treated as if it had not been scattered at all, and so is incorporated into the direct rather than the diffuse downwelling flux. It was estimated by Villefranque (2019) that for scattering by liquid clouds, scattering by angles up to 25° is thereby incorporated into the direct flux. The ECMWF radiation products are computed using delta-Eddington scaling, which should improve the calculation of MRT in this paper, since for estimating the interception of solar radiation by an upright human, it is more appropriate to treat this forward-scattered radiation as if it were travelling directly from the Sun than treating it as diffuse isotropic radiation from the sky. Uncertainty in cloud predictability and other radiation-related parameters, such as albedo, originated also from the model resolution. Even at the finest grid cell size, NWP outputs are a collection of values averaged over a cell, whereas station observations are values collected at one specific point. The difference between NWP and observations increases as the cell size increases, when the terrain is complex and its surface presents changeable roughness and type. The lowest *R*^2^ and the highest bias and RMSE, for instance, are found for Izaña station which is installed in a location (mountain top, high elevation) different from the location of the other stations (flat or hilly, low elevation; Table 2). These findings extend to the global-scale previous research on the uncertainty of NWP radiation fluxes and its implication to MRT calculation that was assessed at selected European study sites (Schreier and al. 2013). With regard to MRT, NWP-based MRT is in good agreement with observed MRT across all stations. It is worth noting that validation metrics are better for MRT than they are for its single radiation inputs. This is because MRT is an aggregate measure, i.e. it incorporates multiple radiation components that can compensate each other’s biases and errors. Future model developments, e.g. in cloud parameterization, are expected to reduce radiation-related uncertainties and thus improve the estimation of MRT from NWP models.

Based on the validation results presented in this study, the globe-wide MRT computed from ECMWF NWP outputs can be used as a proxy for observations. Future studies could further assess NWP-based MRT against observed MRT in different locations and at different spatial scales. Furthermore, the framework here presented is general. It can, for instance, be used to compute MRT in the presence or absence of direct solar radiation (shade). It can be applied on the forecasts of the different radiation components to generate forecasts of MRT. The process could be automated and deliver gridded maps of predicted MRT at the global scale. Maps of MRT could also be generated at the local scale (e.g. in Leroyer et al. 2018) using microclimate models such as ENVI-met and SOLWEIG to simulate spatial variations of thermal comfort in the urban environment (Bruse and Fleer 1998; Lindberg et al. 2008). It should be noted that the NWP-based MRT here presented does not account for urban effects—such as the heat island effect, the thermal emission from building walls and the shadowing of the Sun by buildings—which have been shown to influence human biometeorological comfort in cities (Lindberg et al. 2014). Although some other NWP models include an urban canopy scheme (Masson 2000; Porson et al. 2010), the current ECMWF NWP model does not yet but work is in progress to develop an urban tile that could represent the radiative interaction with streets, walls and roofs (Balsamo et al. 2009; Hogan 2019). When the tile is included into the ECMWF NWP model, urban density and geometry will affect radiation outputs and, as a consequence, the MRT. It should be recalled from Eq. (14) that NWP-based MRT has been computed by setting angle factors equal to 0.5. This has been chosen considering radiation fluxes as coming from two directions, the ground and the sky, and it is commensurate with the data currently available from the ECMWF NWP model. In urban settings, however, the two-direction approach has been shown to underestimate MRT at low Sun elevations as a standing person receives most of the radiation from the sides (Ali-Toudert and Mayer 2007; Thorsson et al. 2007). Applying equations that consider the radiation fluxes coming from six directions (ground, sky and 4 cardinal points; e.g. by Höppe 1992) will help to better capture horizontal fluxes. Until then, the use of microclimate models, supported by field measurements, is critical to downscale the MRT to the urban level (Lindberg et al. 2014). Using angle factors equal to 0.5 may also lead to errors when MRT is calculated in areas with complex orography such as in mountain valleys, where the overall share of the sky is generally less than 50% and the share of the ground more than 50%. Applying angle factors that take this into account is strongly advised.

The relationship between thermal comfort and public health is another potential application for NWP-based MRT. The MRT has been demonstrated as a better predictor of heat-related mortality than air temperature in Stockholm County, Sweden (Thorsson et al. 2014). Maps of MRT will allow future studies to assess the MRT–mortality relationship in other areas of the world, even in those where meteorological stations are not present. Similarly, as one of the parameters used to describe the heat load experienced by the human body, NWP-based MRT could be used to compute reanalysis and forecast maps of thermal stress indices such as the UTCI and investigate the effects of the environment—both radiant and not radiant (e.g. air temperature, humidity and wind speed)—on human health (Pappenberger et al. 2015; Di Napoli et al. 2018, 2019; Vitolo et al. 2019).

## Conclusions

This paper presents a method for the computation of the mean radiant temperature (MRT) at the global scale for a human subject placed in an unobstructed, flat outdoor environment and irradiated by solar and thermal radiation both directly and diffusely.

The proposed method requires (a) the knowledge of radiation fluxes as computed by the numerical weather prediction (NWP) model, i.e. gridded and globe-wide, and (b) the correct handling of the time component over which mean radiation fluxes are accumulated. Equations to account for (b) and average the Sun’s position over the daytime part of the radiation time step are provided.

Using radiation reanalysis data from the ECMWF NWP model, mapped distributions of MRT at the global scale were computed. A comparison against measurements from the World Radiation Monitoring Center–Baseline Surface Radiation Network shows a good agreement for NWP-based MRT ( *R*^2^ greater than 0.88; average bias and RMSE equal to 0.42 °C and 5.99 °C, respectively). This suggests a potential use of NWP-based MRT as proxy for observation in applications studies, e.g. human thermal comfort, where the radiant environment represented by MRT plays an important role.
